# Identification of *Lactobacillus plantarum *genes modulating the cytokine response of human peripheral blood mononuclear cells

**DOI:** 10.1186/1471-2180-10-293

**Published:** 2010-11-16

**Authors:** Saskia van Hemert, Marjolein Meijerink, Douwe Molenaar, Peter A Bron, Paul de Vos, Michiel Kleerebezem, Jerry M Wells, Maria L Marco

**Affiliations:** 1 TI Food & Nutrition, Nieuwe Kanaal 9A, 6709PA, Wageningen, The Netherlands; 2 NIZO food research, P.O. Box 20, 6710 BA Ede, The Netherlands; 3 Host-Microbe Interactomics, Animal Sciences, Wageningen University, P.O. Box 338, 6700 AH Wageningen, The Netherlands; 4 Vrije Universiteit Amsterdam, Faculty of Earth and Life Sciences, De Boelelaan 1085, 1081 HV Amsterdam, The Netherlands; 5 Kluyver Centre for Genomics of Industrial Fermentation, P.O. Box 5057, 2600 GA, Delft, The Netherlands; 6 Immunoendocrinology, Pathology and Medical Biology, University Medical Centre Groningen, Hanzeplein 1, 9700 RB Groningen, The Netherlands; 7 Laboratory of Microbiology, Wageningen University, Dreijenplein 10, 6703 HB Wageningen, The Netherlands; 8 Department of Food Science and Technology, One Shields Avenue, Davis, CA 95616, USA

## Abstract

**Background:**

Modulation of the immune system is one of the most plausible mechanisms underlying the beneficial effects of probiotic bacteria on human health. Presently, the specific probiotic cell products responsible for immunomodulation are largely unknown. In this study, the genetic and phenotypic diversity of strains of the *Lactobacillus plantarum *species were investigated to identify genes of *L. plantarum *with the potential to influence the amounts of cytokines interleukin 10 (IL-10) and IL-12 and the ratio of IL-10/IL-12 produced by peripheral blood mononuclear cells (PBMCs).

**Results:**

A total of 42 *Lactobacillus plantarum *strains isolated from diverse environmental and human sources were evaluated for their capacity to stimulate cytokine production in PBMCs. The *L. plantarum *strains induced the secretion of the anti-inflammatory cytokine IL-10 over an average 14-fold range and secretion of the pro-inflammatory cytokine IL-12 over an average 16-fold range. Comparisons of the strain-specific cytokine responses of PBMCs to comparative genome hybridization profiles obtained with *L. plantarum *WCFS1 DNA microarrays (also termed gene-trait matching) resulted in the identification of 6 candidate genetic loci with immunomodulatory capacities. These loci included genes encoding an *N*-acetyl-glucosamine/galactosamine phosphotransferase system, the LamBDCA quorum sensing system, and components of the plantaricin (bacteriocin) biosynthesis and transport pathway. Deletion of these genes in *L. plantarum *WCFS1 resulted in growth phase-dependent changes in the PBMC IL-10 and IL-12 cytokine profiles compared with wild-type cells.

**Conclusions:**

The altered PBMC cytokine profiles obtained with the *L. plantarum *WCFS1 mutants were in good agreement with the predictions made by gene-trait matching for the 42 *L. plantarum *strains. This study therefore resulted in the identification of genes present in certain strains of *L. plantarum *which might be responsible for the stimulation of anti- or pro-inflammatory immune responses in the gut.

## Background

Metagenomics and host-microbe molecular interaction studies are rapidly expanding our understanding of the indigenous gut microbiota and the contributions of microbes to human health [1,2]. These efforts are complementary to the numerous reports describing health benefits associated with the ingestion of probiotic bacteria [3,4]. Probiotics are living microorganisms which confer health effects on the host when administered in sufficient amounts [5]. Strains of *Lactobacillus *and *Bifidobacterium *are the most commonly applied probiotics in food products. Members of these genera are residents of the human intestine and have a long history of safe use in foods and beverages. Health benefits conferred by probiotics can be specific to the gastrointestinal tract (e.g. protection against intestinal inflammation or enteric pathogens) or occur at peripheral mucosal sites in the human body (e.g. prevention of allergy or dermatitis) [6].

There is substantial evidence that an important mechanism by which probiotics provide health benefits is through modulation of immune functions [7-11]. Differences among probiotic strains to stimulate immune cells towards pro- and anti-inflammatory responses have been shown in studies measuring cytokine production *in vitro *[7-11]. These comparisons have resulted in the identification of strains inducing similar responses *in vivo*. For example, ratios of IL-10 to IL-12 produced by Peripheral Blood Mononuclear Cells (PBMCs) in response to different probiotics *in vitro *were correlated to their protective capacity in a mouse model of colitis [10]. Similarly, recent studies on the mechanisms of probiotics highlight their effects on epithelial barrier function via Toll-like receptor 2 signaling and the generation of regulatory dendritic cells and regulatory CD4+Foxp3+ T cells in peripheral tissues [12,13]. The latter mechanism is linked to the administration of a collection of five strains which induced a high IL-10/IL-12 ratio in co-culture with immune cells [12]. Administration of these strains was shown to have a therapeutic effect in experimental mouse models of inflammatory bowel disease, atopic dermatitis, and rheumatoid arthritis and was associated with enrichment of CD4(+)Foxp3(+) Tregs in the inflamed regions [12].

The cell products of probiotics that are responsible for modulation of cytokine induction are largely not known but might involve modifications of some of the known Microbe Associated Molecular Patterns (MAMPs) such lipoteichoic acids (LTA) [14-16] and (lipo)proteins localized on the bacterial cell surface [17] which interact with Toll-like receptors. Additionally cell-surface associated bacterial glycosylated proteins or exopolysaccharides [18] may interact with other host pattern recognition receptors including the C-type lectins and scavenger receptors found on antigen presenting cells [19]. These extracellular and secreted products produced by probiotic cells are the likely targets for strain-dependent interactions with host cells and have been the focus of several recent reviews [6,20,21].

Certain strains of *Lactobacillus plantarum *are marketed as probiotics and reported to confer various health effects including immunomodulation [22]. The genome sequence of *L. plantarum *strain WCFS1 is known [23] and extensive bioinformatics tools [24,25], molecular models [26], and a database of genome hybridization profiles [27,28] are available for this organism. It is a single colony isolate of strain NCIMB8826, which was shown to survive gastrointestinal passage after oral administration to healthy volunteers [29]. Global gene expression profiling of *L. plantarum *WCFS1 in the intestinal contents of the human gut and conventionally-raised and germ-free mice has shown that this organism adapts for growth *in vivo *by modification of its cell-surface composition and metabolism in a diet-dependent manner [30-34]. Human duodenal transcriptional response profiles have also been obtained in response to ingestion of *L. plantarum *WCFS1 [35,36]. Notably, exponential phase and stationary phase *L. plantarum *WCFS1 cells elicited distinct human duodenal transcript profiles which appeared to mainly result from differential modulation of canonical NF-κβ-dependent signaling pathways associated with immune tolerance [35].

The aim of the present study was to identify genes involved in immunomodulation by *L. plantarum *WCFS1. The IL-10 and IL-12 cytokine levels elicited by peripheral blood mononuclear cells (PBMCs) upon stimulation with *L. plantarum *WCFS1 and 41 other *Lactobacillus plantarum *strains were determined. We compared the IL-10 and IL-12 stimulating phenotypes of each strain to its genome composition determined by comparative genome hybridization (CGH) to identify candidate *L. plantarum *genes with the capacity to affect cytokine production in PBMCs. The immunomodulatory potential of these gene products was confirmed for *L. plantarum *WCFS1 gene deletion mutants and found to be dependent on the growth-phase of the *L. plantarum *cultures.

## Results

### Immunomodulation of PBMCs is a variable phenotype in *L. plantarum*

**Figure 1:**
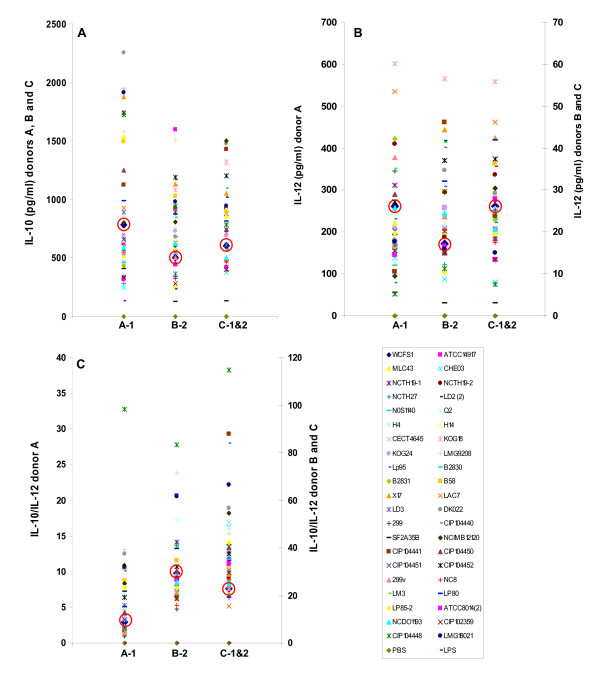
**Cytokine secretion by PBMCs after 24 h co-culture with *L. plantarum *strains**. IL-10 (A) and IL-12 (B) production and the IL-10/IL-12 ratio (C) by peripheral blood mononuclear cells (PBMCs) derived from blood of 3 different healthy donors after stimulation with 42 different *L. plantarum *strains harvested in stationary-phase. The *L. plantarum *strains grown and prepared on separate days constitute set 1 and set 2. PBMCs isolated from donor A were inoculated with *L. plantarum *culture set 1 (A-1) and PBMCs from donor B were inoculated with the *L. plantarum *replicate set 2 (B-2). PBMCs from Donor C received both sets of cultures and the mean of the IL-10 and IL-12 amounts induced by these cultures is shown. Each symbol represents a different *L. plantarum *strain or the PBS or LPS controls.

A total of 42 *L. plantarum *strains from distinct (fermented) food, environmental, and gastrointestinal sources (Table 1 and [27,28]) were investigated for their capacities to stimulate PBMCs to produce the cytokines IL-10 and IL-12. Comparisons of cytokine amounts induced among different donors in response to the *L. plantarum *strains showed that the *L. plantarum *cultures induced a similar range of IL-10 but up to 10-fold different levels of IL-12 (Figure 1). This result is agreement with previous studies showing that PBMCs respond differently depending on the donor from which the cells were isolated [37]. However, the capacity of individual *L. plantarum *strains to induce cytokines production in PBMCs was similar among the different donors relative to the other strains tested. For example, *L. plantarum *KOG18 consistently induced the highest amounts of IL-12 whereas strain CIP104448 induced the highest ratios of IL-10 to IL-12. Collectively, the 42 *L. plantarum *strains induced, on average, IL-10 and IL-12 in PBMCs over a 14- and 16 - fold range, respectively, and IL-10/IL-12 ratios over a 13.5 - fold range (Figure 1). Strain WCFS1 induced relatively low IL-10 amounts (between 440 and 780 pg/ml), moderate amounts of IL-12 (between 20 and 260 pg/ml), and consequently a moderate to low IL-10/IL-12 ratio (bottom quartiles) compared with the other strains (Figure 1).

**Table 1:** L. plantarum strains selected for genotyping and screening for immunomodulatory capacity.

Strain	**Strain ID**^**a**^	Isolation source	Geographical origin
WCFS1	NIZO1836	Human saliva	n.a.
LP80	NIZO2263	Silage	n.a.
Lp95	NIZO2814	Wine red grapes	Italy
CIP102359	CIP102359	Human spinal fluid	France
ATCC8014	NIZO2726	Maize ensilage	n.a.
LD3	NIZO2891	Radish pickled	Vietnam
CHEO3	NIZO2457	Pork pickled sour sausage	Vietnam
LD2	NIZO2535	Orange fermented	Vietnam
BLL(EI31)	NIZO2830	n.a.	Not known
CIP104452	NIZO2259	Human tooth abscess	France
CECT221(24Ab04)	NIZO2831	Grass silage	United States
LM3	NIZO2262	Silage	n.a.
NCTH27	NIZO2494	Pork pickled sour sausage	Vietnam
NCDO1193	NCDO1193	Vegetables	n.a.
LMG9208	NIZO2806	Sauerkraut	United Kingdom
ATCC14917	NIZO2896	Cabbage pickled	Denmark
NOS140	NIZO2741	Cabbage kimchi	Japan
299	NIZO1837	Human colon	United Kingdom
N58	NIZO2855	Pork pickled sour sausage	Vietnam
X17	NIZO2877	Hot dog	Vietnam
299v/DSM9843	NIZO2260	Human intestine	United Kingdom
MLC43	NIZO2029	Raw cheese with rennet	Italy
LAC7	NIZO2889	Banana fermented	Vietnam
LP85-2	NIZO2264	Silage	France
NCTH19-1	NIZO2484	Pork pickled sour sausage	Vietnam
NCTH19-2	NIZO2485	Pork pickled sour sausage	Vietnam
NC8	NIZO2261	Grass silage	Sweden
KOG24	NIZO2802	Cheese	Japan
KOG18	NIZO2801	Turnip pickled	Japan
LMG18021	NIZO3400	Milk	Senegal
Q2	NIZO2753	Sourdough fermented	Italy
SF2A35B	NIZO1839	Sour cassava	South America
CIP104451	NIZO2258	Human urine	France
CIP104450	NIZO2257	Human stool	France
CIP104448	CIP104448	Human stool	France
DKO22	NIZO2897	Sour cassava	Nigeria
H14	NIZO2766	Sourdough fermented	Italy
H4	NIZO2757	Sourdough fermented	Italy
CECT4645	NIZO2776	Cheese	n.a.
CIP104441	NIZO2256	Human stool	France
CIP104440	NIZO1838	Human stool	France
NCIMB12120	NIZO1840	Cereal fermented (Ogi)	Nigeria

n.a. not available

^**a **^See references [27,28] for comparative genome hybridization analyses of these strains.

### Identification of candidate genes involved in immunomodulation

To identify candidate *L. plantarum *genes involved in the modulation of the immune response, Random Forest models [38] were used to compare *L. plantarum *CGH profiles with the relative amounts of IL-10 and IL-12 and IL-10/IL-12 ratios induced by the strains in co-culture with PBMCs (Figure 1). PBMCs from different donors incubated with replicate *L. plantarum *cultures were used for these models to take into account the levels of variation in cytokine production. Comparisons of *L. plantarum *strain genotype to the IL-10-stimulating capacities resulted in the identification of 6 different chromosomal loci and a total of 13 genes that might influence IL-10 production (Table 2). In comparison, concise correlations between *L. plantarum *CGH profiles and IL-12 amounts were not found. One of the genes correlated with IL-10 amounts was *L. plantarum *WCFS1 lp_1953. *L. plantarum *strains harboring this gene stimulated the production of IL-10 in 1.6-fold higher amounts, on average, compared to *L. plantarum *strains for which this gene was absent. Lp_1953 encodes a hypothetical intracellular protein of unknown function [25]. The remaining five genes with putative roles in IL-10 modulation comprise a putative 5 gene operon (lp_2647 to lp_2651) encoding Pts19ADCBR, an N-acetyl-galactosamine/glucosamine phosphotransferase system (PTS). Strains harboring these genes were associated with induction of lower amounts of IL-10 by PBMCs.

**Table 2:** L. plantarum genes with putative roles in modulating PBMC cytokine production.

Genes(s)	**Gene number**^**a**^	Product	**Percent of strains with the gene(s)**^**b**^	**Gene-dependent contribution to cytokine stimulation**^**c**^	
lp_1953	lp_1953	Hypothetical protein	48	IL-10	1.6-fold ↑
*pts19ADCBR*	lp_2647-2651	N-galactosamine PTS, EIIADCB and transcription regulator, GntR family	33	IL-10	1.7-fold ↓
*plnEFI*	lp_0419-0422	Immunity protein PlnI	81-85	IL-10/IL-12	1.7-fold ↓
		Bacteriocin-like peptide PlnF			
		Bacteriocin-like peptide PlnE			
*plnG*	lp_0423	ABC transporter	88	IL-10/IL-12	1.8-fold ↓
*lamB*	lp_3582	Accessory gene regulator protein	43	IL-10/IL-12	1.3-fold ↓
prophage P2b 1 & 21	lp_2460	Prophage P2b protein 21	38	IL-10/IL-12	1.5-fold ↑
	lp_2480	Prophage P2b protein 1, integrase			

^**a **^Gene number on the *L. plantarum *WCFS1 chromosome [23].

^**b **^Percentage of *L. plantarum *strains containing the gene according to CGH [27,28].

^**c **^Gene-trait matching importance measures (in parentheses) and predicted effects of the gene(s) on the variable and average magnitude and direction (higher or lower) of IL-10 and IL-10/IL-12 amounts.

Comparisons between *L. plantarum *strain-specific CGH profiles and IL-10/IL-12 ratios from PBMCs resulted in the identification of four *L. plantarum *WCFS1 loci which correlated with IL-10/IL-12 values (Table 2). *L. plantarum *WCFS1 *plnEFI *and *plnG *(lp_419-423) and *lamB *(lp_3582) were most commonly present in strains stimulating low IL-10/IL-12 ratios. These genes are under the control of the auto-inducing peptide (AIP)-based quorum sensing (QS) two-component regulatory systems (QS-TCSs) found in *L. plantarum *[39,40]. The genes *plnEFI *and *plnG *encode two bacteriocin peptides, a bacteriocin immunity protein, and an ATP - Binding Cassette (ABC) transporter [23,41]. The *lamB *is the first gene in the *L. plantarum lamBDCA *operon and shows 30% amino acid identity to the *S. aureus *AgrD-processing protein AgrB required for AIP modification and export [39]. The other *L. plantarum *genes associated with specific IL-10/IL-12 ratios are lp_2460 and lp_2480 coding for prophage R-Lp3 remnant proteins P2b protein 21 and 1, respectively [23]. These genes are conserved among *L. plantarum *strains stimulating high IL-10/IL-12 ratios in PBMCs. The functions of prophage R-Lp3 and other complete prophages in *L. plantarum *WCFS1 genome are not known [42]. Because the different prophages found in *L. plantarum *WCFS1 share high levels of sequence homology and potential functional redundancy [42], these genes were not examined further.

### Verification of the roles of the candidate genes in immunomodulation

To validate the influence of the candidate *L. plantarum *genes on PBMC cytokine responses, lp_1953, *pts19ADCBR, plnEFI*, and *plnG *deletion mutants were constructed for *L. plantarum *WCFS1. A previously constructed *L. plantarum *WCFS1 *lamA *(lp_3580)*lamR *(lp_3087) double mutant was used to examine the potential roles of the *lamBCDA *QS-TCS on PBMCs. This strain was selected because *lamA *and *lamR *encode the response regulators of the 2 TCS (*lamBCDA *and *lamKR*) regulating the expression of the LamD AIP in *L. plantarum *WCFS1 [40]. In the Δ*lamA *Δ*lamR *mutant, expression levels of *lamB *and the other genes in this operon were at 5% of the levels found in wild-type cells [40].

**Figure 2:**
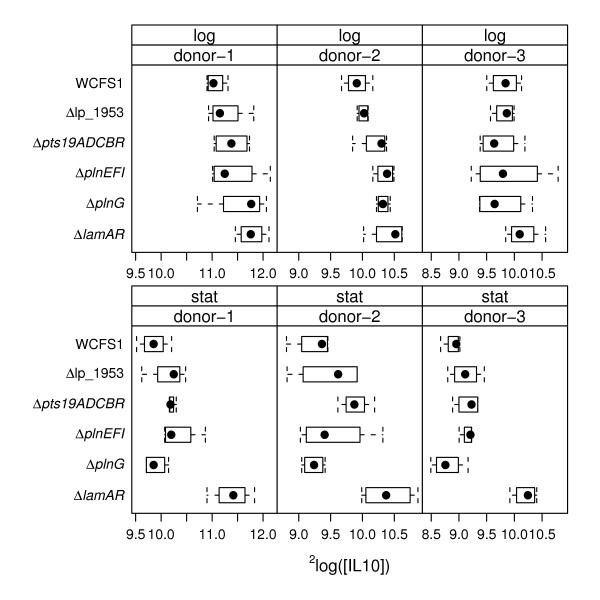
**Boxplots of IL-10 amounts produced by PBMCs in response to *L. plantarum *wild-type and mutant cells**. ^2^Log transformed IL-10 amounts induced by exponential and stationary phase *L. plantarum *cells are shown. The dots indicate the median value, the boxes indicate first and third quartile, and the whiskers extend to outlying data points for a total of 12 measurements (3 PBMC donors were measured using 4 replicate cultures of each *L. plantarum *strain).

**Figure 3:**
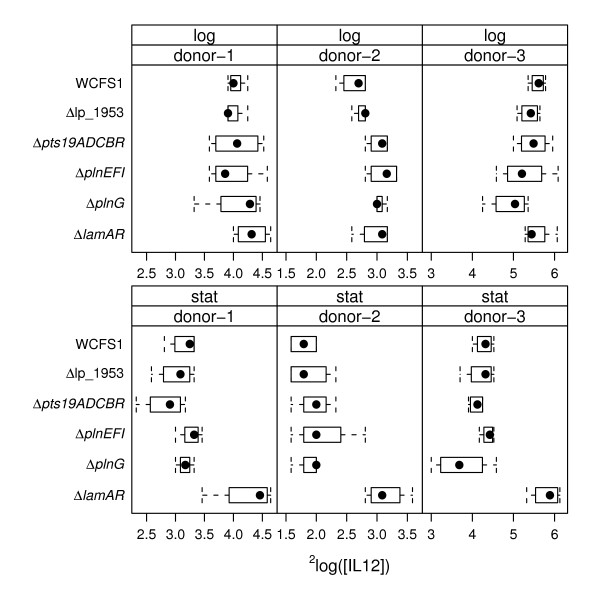
**Boxplots of IL-12 amounts produced by PBMCs in response to *L. plantarum *wild-type and mutant cells**. ^2^Log transformed IL-12 amounts induced by exponential and stationary phase *L. plantarum *cells are shown. The dots indicate the median value, the boxes indicate first and third quartile, and the whiskers extend to outlying data points for a total of 12 measurements (3 PBMC donors were measured using 4 replicate cultures of each *L. plantarum *strain).

Wild-type and mutant *L. plantarum *WCFS1 cells harvested in the stationary- and exponential phases of growth were examined for their capacity to stimulate IL-10 and IL-12 in PBMCs. Overall, among the donors examined, IL-10 and IL-12 were produced in response to *L. plantarum *at levels between 500 to 4500 pg/ml and 3 to 68 pg/ml, respectively (shown as log_2 _values in Figure 2 and 3). Notably, exponential cultures of wild-type *L. plantarum *WCFS1 and most mutant strains stimulated PBMCs to secrete higher amounts of IL-10 and IL-12 than stationary-phase cells (Figure 2 and 3).

**Figure 4:**
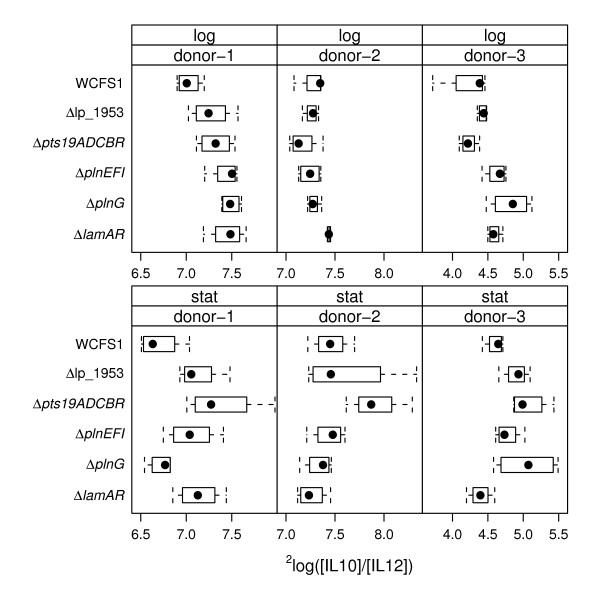
**Boxplots of IL-10/IL-12 amounts produced by PBMCs in response to *L. plantarum *wild-type and mutant cells**. ^2^Log transformed IL-10/IL -12 ratios induced by exponential and stationary phase *L. plantarum *cells are shown. The dots indicate the median value, the boxes indicate first and third quartile, and the whiskers extend to outlying data points for a total of 12 measurements (3 PBMC donors were measured using 4 replicate cultures of each *L. plantarum *strain).

*L. plantarum *strains harboring the *plnEFI, plnG *or *lamB *loci were associated with the stimulation of lower IL-10/IL-12 ratios by *L. plantarum *in the PBMC assay (Table 2). In agreement with the gene-trait correlations, the *plnEFI, plnG*, and *lamA lamR *deletion mutants of strain WCFS1 induced higher IL-10/IL-12 ratios than the wild-type strain (Figure 4 and Table 3). However, the effects of the *plnEFI *deletion on cytokine induction in different donors was not highly significant compared to wild-type *L. plantarum *when the p value was adjusted for multiple hypothesis testing (adjusted (adj.) p value = 0.071) (Figure 4 and Table 3). Mutants deficient in the ABC- transporter *plnG *induced significantly higher cytokine ratios compared with *L. plantarum *wild-type cells (Figure 4 and Table 3). These differences were observed only for wild-type and mutant cells harvested during exponential phase growth (adj. p value = 0.005). Immunomodulation of the Δ*lamA *Δ*lamR *mutant was also substantially different compared to wild-type *L. plantarum *WCFS1. The Δ*lamA *Δ*lamR *mutant induced significantly higher IL-10/IL-12 ratios (adj. p value = 0.016) and IL-12 (adj. p value < 0.001) and IL-10 (adj. p value < 0.001) amounts in PBMCs (Table 3). These effects were partially dependent on the growth-phase of the *L. plantarum *cells. IL-10/IL-12 ratios and IL-10 amounts induced by wild-type and mutant cells were significantly different when exponential phase cultures were used in the PBMC assay, whereas IL-10 and IL-12 amounts also differed when stationary-phase cells were examined (Figure 2, 3, 4 and Table 3).

**Table 3:** Relative differences in cytokine amounts between L. plantarum WCFS1 wild-type and deletion mutants.

		IL-10^c^			IL-12			IL-10/IL-12		
Mutant **comparison**^**a**^	Growth **phase**^**b**^	value	p-value	adj. p- value	value	p-value	adj. p- value	value	p-value	adj. p- value
**lp_1953**	log	0.097	0.461	0.830	-0.041	0.775	0.825	0.138	0.161	0.803
	stat	0.253	0.057	0.228	-0.043	0.761	0.825	0.296	0.003	0.024 *
***pts19ADCBR***	log	0.164	0.216	0.647	0.106	0.458	0.825	0.058	0.556	0.923
	stat	0.396	0.004	0.031 *	-0.131	0.371	0.825	0.529	0.000	0.000 ***
***plnEFI***	log	0.287	0.031	0.176	0.032	0.825	0.825	0.255	0.010	0.071
	stat	0.344	0.010	0.071	0.174	0.225	0.825	0.170	0.084	0.507
***plnG***	log	0.280	0.035	0.176	-0.070	0.625	0.825	0.350	0.000	0.005 **
	stat	-0.028	0.830	0.830	-0.146	0.307	0.825	0.118	0.230	0.921
***lamA lamR***	log	0.511	0.000	0.001 ***	0.199	0.165	0.825	0.312	0.002	0.016 *
	stat	1.331	0.000	0.000 ***	1.321	0.000	0.000 ***	0.009	0.923	0.923

^**a **^*L. plantarum *WCFS1 deletion mutant measured in the PBMC assay.

^**b **^Phase of growth from which *L. plantarum *cells were harvested (log = exponential phase; stat = stationary phase).

^**c **^The value is the average difference in ^2^Log cytokine amounts induced by wild-type *L. plantarum *and mutant cells harvested in the same phase of growth (log or stat). A positive value indicates an increase in IL-10 levels produced by PBMCs in response to mutant *L. plantarum *compared to the wild-type cells. Calculations of t-test p-values and adjusted (adj.) p-values are described in the text (Materials and Methods). * (0.01 < p < 0.05); ** (0.002 < p < 0.01); *** (p < 0.002) for the adj. p-values.

In agreement with the gene trait matching correlations, the Δ*pst19ADCBR *mutant induced significantly higher amounts of IL-10 than wild-type *L. plantarum *(adj. p value = 0.031) (Figure 2 and Table 3). Similarly, the IL-10/IL-12 ratio was significantly higher (p < 0.001) upon stimulation with *L. plantarum *Δ*pst19ADCBR *compared with the parental strain (Figure 4 and Table 3). *L. plantarum *strains harboring lp_1953 were also predicted to induce higher IL-10 production levels compared with strains lacking this gene. However, the *L. plantarum *lp_1953 deletion mutant stimulated equivalent amounts of IL-10 and somewhat higher IL-10/IL -12 ratios (adj. p value = 0.024) relative to wild-type *L. plantarum *WCFS1 (Figure 4 and Table 3). Although the lp_1953 mutant induces a modest, yet significantly different, IL-10/IL-12 response relative to the parental strain, these results are not in agreement with the immunomodulatory effects predicted for this gene.

In summary, of the 5 mutants tested here, three (Δ*lamA *Δ*lamR*, Δ*pst19ADCBR*, and Δ*plnG*) significantly affected the immune response of PBMCs in different donors according to the phenotypes predicted from the gene-trait matching data (Table 2). The *plnEFI *mutant also affected the immune response in the predicted manner but this was not significant considering the adjusted p value. The Δ*lamA *Δ*lamR *mutant conferred the largest differences on the induction of IL-10 and IL-12 and the IL-10/IL-12 ratio by *L. plantarum *(Table 3).

## Discussion

This study demonstrated the diverse capacities of *L. plantarum *strains to stimulate cytokine production in human PBMCs and confirmed the contributions of specific *L. plantarum *genes to modulate these responses. Forty-two *L. plantarum *strains induced PBMCs to secrete IL-10 over an average 14-fold range. This range was similar to IL-10 amounts stimulated by 7 *Bifidobacterium longum *strains [43] and the 10 to 15-fold differences in cytokine amounts induced in PBMCs by multiple *Lactobacillus *and *Bifidobacterium *species [7-11]. Moreover, we found that variation in IL-10 and IL-12 amounts and IL-10/IL-12 ratios induced by the distinct *L. plantarum *strains was higher than reported previously [44]. This result was probably due to the analysis of more strains in the present study (42 versus 3), which were isolated from diverse environmental niches encompassing a greater genetic and phenotypic diversity of the *L. plantarum *species. Such strain-specific differences should therefore be taken into consideration when selecting a probiotic *Lactobacillus *culture for health conditions which are dependent on modulating immunity such as in the prevention of allergy, eczema, or inflammatory bowel disease.

To identify *L. plantarum *genes with roles in modulating immune cell responses, *L. plantarum *genetic diversity was correlated with strain-specific capacities to induce cytokines in PBMCs. Genes with putative contributions to the observed PBMC responses were further investigated in *L. plantarum *WCFS1. A similar gene-trait matching approach previously resulted in the identification of a *L. plantarum *mannose-specific adhesin (Msa) [45] and genes which modulate dendritic cell responses [46]. Although the gene-trait matching approach has been successful, it should be recognized that only a subset of immunomodulatory cell components produced by *L. plantarum *was likely identified here. Firstly, the identified immunomodulatory genetic loci were restricted to genes in the *L. plantarum *WCFS1 reference strain genome. Secondly, genes with high levels of sequence conservation such that they are not distinguished by CGH (presence versus absence, rather than minor sequence variations) might be excluded from detection. For example, *L. plantarum *highly conserved LTA biosynthesis and modification genes known to have established effects on mammalian immunity were not found in this biodiversity-based gene-trait matching approach. Finally, genetic assessments do not take into account strain-specific variations in gene expression, translation, or post-translational modification of proteins with immunomodulatory effects.

Despite these limitations and the considerable variation in the production of cytokines by PBMCs from different donors, the present study demonstrated that gene-trait matching is also suitable for the identification of genes that affect cytokine levels in the mixture of immune cells collectively termed PBMCs. The products of AIP-based QS-TCSs and the N-acetyl-galactosamine/glucosamine phosphotransferase system identified here might constitute a new class of bacterial cell products which are recognized by host receptors. The findings are significant because these genes were identified using intact cells which likely have multiple interactions with immune cells such that single genes only confer incremental effects.

*L. plantarum *WCFS1 *lamB*, a processing/export protein of the AIP-based QS-TCS LamBDCA [47], was correlated with immunomodulation of PBMCs. LamB, a transmembrane protein, is under the control of two response regulators *lamA *and *lamR *[40]. A *L. plantarum *Δ*lamA *Δ*lamR *mutant investigated in this study was found to induce PBMCs to secrete significantly higher amounts of the cytokines IL-10 and IL-12. In a previous report, global transcript profiling of the *lamA lamR *deletion mutant showed that the *lamBDCA *system is auto-regulated and controls the production of several surface-associated proteins, stress-associated functions, and surface polysaccharides [40]. Higher amounts of surface polysaccharides produced by *L. plantarum *Δ*lamA *Δ*lamR *decreased the biofilm-forming capacity of the mutant strain [40]. Polysaccharides produced by some *Lactobacillus *species are known for their immunomodulatory effects either by direct interactions with immune cells or by shielding MAMPs on the bacterial cell surface from detection by the immune system [18,48,49]. Therefore the observed PBMC IL-10/IL-12 ratios for *L. plantarum *might either be mediated directly through the LamBDCA system and the cognate secreted peptide, or indirectly through cell products (e.g., polysaccharides) under the control of this regulatory system. The latter is supported by the genetic similarities between LamBDCA and the *Staphylococcus aureus agr *system, an AIP-based QS-TCS which controls the evasion of innate host defenses by *S. aureus *through the production of secreted peptides and proteases [50].

The plantaricin biosynthesis pathway of *L. plantarum *WCFS1 is also controlled by an AIP-based QS-TCS [47] and genes required for plantaricin production and transport contributed to *L. plantarum *effects on PBMCs. Plantaricin is a bacteriocin composed of two small secreted peptides (*plnE *and *plnF*) which destabilize the integrity of the plasma membrane of susceptible cells [51]. *L. plantarum *strains harboring *plnEF *and *plnI *encoding a plantaricin immunity protein, and/or *plnG *encoding a membrane bound ABC-transporter induced PBMCs to secrete IL-10 and IL-12 in amounts that yielded lower IL -10/IL-12 ratios (Table 2). Similarly, wild-type *L. plantarum *WCFS1 conferred lower IL-10/IL-12 ratios compared to the *plnEFI *and *plnG *deletion mutants, although this was significant only for the *plnG *mutant (p = 0.005) and not the mutant lacking *plnEFI *(p = 0.071). The identification of the AIP plantaricin is intriguing because human antimicrobial peptides such as defensins secreted in the gut are known to modulate immune responses [52,53] and suggest that antimicrobial peptides of bacterial origin might have similar capacities. These findings are also compatible with a recent study showing that plantaracins can modulate dendritic cell responses [46]. Moreover, several independent studies showed that *L. plantarum *WCFS1 genes involved plantaricin biosynthesis and activity, including *plnI *and *plnF*, are induced in the mouse gut [30-32], thereby indicating that plantaricin production is active in the intestine where it might come into contact with mucosal immune cells.

Another of the confirmed genes with immunomodulatory capacities was the *pts19ADCBR *locus coding for a cell membrane-associated N-acetyl-galactosamine/glucosamine phosphotransferase system. The relevance of the *pts19ADCBR *genes in adaptation to the intestinal ecosystem was also demonstrated by their higher expression levels in the intestine of conventionally-raised and germ-free mice [31,32]. Moreover, in *Lactobacillus johnsonii*, a putative mannose phosphotransferase gene locus with 43% amino acid identity to the *L. plantarum *WCFS1 *pts19ADCBR *cluster was found to be important for long term persistence *in vivo *[54]. Although the regulatory signals for expression of these genes are unknown, immunomodulatory effects conferred by Pts19ADCBR might influence the ability of *L. plantarum *to modify the intestinal environment for survival in the gut.

Cytokine profiles of the lp_1953 deletion mutant were not in agreement with the IL-10 stimulating capacity predicted for this gene by gene-trait matching. This result exemplifies the need for mutation analysis to confirm gene-trait predictions, which are likely to encompass false-positive associations. A similar conclusion was drawn during the identification of the *L. plantarum *Msa gene [45]. Moreover, the product of lp_1953 is predicted to be intracellular, which contrasts the predicted subcellular location of all other genes examined here (secreted or cell envelope associated) [24,25]. This finding supports the notion that surface-localized proteins or components are the most likely candidate-participants in host-microbe interactions [49,55]. Thus far, the majority of the known immunomodulating MAMPs known for lactobacilli are extracellular or cell surface associated products such as LTA, exopolysaccharides, and peptidoglycan, although intracellular CpG-containing oligodeoxynucleotides (ODNs) produced by some lactobacilli are able to induce IL-10 production in immune cells [21,49]. These MAMPs are recognized by specific Pattern Recognition Receptors (PRRs) such as Toll-like receptors (TLRs) and nucleotide oligomerization domain (NOD)-like receptors [21]. To identify the mechanisms underlying the effects of AIP-based QS-TCSs and the N-acetyl-galactosamine/glucosamine phosphotransferase system on immune cells, the cellular products encoded by the genes in these pathways should be investigated to identify the specific cell types among the PBMCs, which include lymphocytes, monocytes and macrophages, that recognize these compounds as well as the specific mechanisms leading to altered cytokine production.

Comparisons of mutant and wild-type *L. plantarum *WCFS1 cells included examination of the effects of culture growth phase on the stimulation of PBMCs. Exponential- and stationary-phase *L. plantarum *WCFS1 cultures were evaluated because the growth phase of probiotic cells was previously shown to influence the immune responses to probiotic bacteria *in vitro *[56-59] and *in vivo *[35]. Using human PBMCs, we found significant growth-phase dependent differences in the immunomodulatory capacities of the wild-type and mutant *L. plantarum *cultures. Collectively, the exponential-phase *L. plantarum *WCFS1 cultures stimulated higher absolute amounts of IL-10 and IL-12 and hence appear to induce heighted immune responses by PBMCs compared with stationary-phase cells. Notably, this result was not due to extensive *L. plantarum *growth because antibiotics were added to the PBMC growth medium to prevent bacterial overgrowth which would generate artifacts from acidification of the medium causing PBMC cell stress or death. Moreover, intact and lysed *L. plantarum *strains cells collected from the exponential and stationary phase of growth do not show striking differences in their TLR9 signaling activity and there was not a clear trend among all strains tested (personal observation, M. Meijerink and J. M. Wells). Therefore the higher amounts of cytokines induced by exponential phase bacteria are unlikely to be caused by differential cell lysis resulting in the release of intracellular CpG DNA, a known MAMP recognized by TLR9.

Comparisons of wild-type and mutant *L. plantarum *cultures also showed growth-phase dependent effects. The IL-10 amounts and IL-10/IL-12 ratios induced by the *pts19ADCBR *deletion mutant were significantly different from wild-type *L. plantarum *WCFS1 for only the stationary-phase cultures. Stationary-phase cells of the Δ*lamA *Δ*lamR *mutant also induced significantly higher amounts of IL-10 and IL-12 in compared with *L. plantarum *WCFS1 harvested at the same growth phase. However, differences between IL-10/IL-12 ratios induced by Δ*lamA *Δ*lamR *and wild-type cell differed only for exponential phase cultures. This result might have been partially due to the extensive alterations in expression of *L. plantarum *Δl*amA *Δ*lamR *in actively growing cultures [39], such that differences in expression of *lamBDCA *and *lamKR *regulated genes might have influenced the ability of the exponential-phase *L. plantarum *cells to stimulate different PBMC IL -10/IL -12 ratios. A similar result was found for the comparisons of *L. plantarum plnG *(and *plnEFI*), the other 2 TCS system examined, although the specific growth-phase-dependent modifications of the plantaricin system on cytokine production in PBMCs is not presently known.

## Conclusions

The present study compared the genetic and phenotypic diversity of *L. plantarum *WCFS1 to identify cell components of this species with the capacity to modulate human PBMC responses. We successfully identified several *L. plantarum *WCFS1 genes that are associated with the production of anti- and pro-inflammatory cytokines by PBMCs and established that the immune response to *L. plantarum *can be significantly altered by the deletion of specific *L. plantarum *cell surface proteins. The increased IL-10/IL-12 ratios of the *L. plantarum *mutants indicate that these cultures would be more protective against intestinal inflammation compared with wild-type cells. These effects might be mediated by the down-regulation of local inflammatory responses through various subsets of T cells producing a collection anti-inflammatory cytokines. As a result of this study, strain selection for protection against intestinal inflammation might include screening for strains lacking the LamB, PlnG, or Pts19 homologs or by modifying culture growth conditions or food delivery matrices to minimize the expression of these genes *in vivo*. Such studies are required to distinguish between health effects conferred by individual probiotic strains and to develop methods to ensure that probiotic cells express host-modulatory cell products at the appropriate level and time in food products and the human gut.

## Methods

### Bacterial strains

Immune assays and genetic analysis was performed on a total of 42 *L. plantarum *strains with distinct phenotypic profiles [27,28] (Table 1). Comparative genome hybridization (CGH) of these strains was performed previously [27,28]. For immunoprofiling, the *L. plantarum *strains were grown at 37°C in Mann-Rogusa Sharpe (MRS) broth (Merck, Darmstadt, Germany) until mid-exponential (optical density (OD) 600 nm = 1) phase or stationary phase (24 h after the start of the culture, OD600 nm = 7.6 ± 1.1) [60]. The exponential and stationary phase cells were washed twice in phosphate buffered saline (PBS) at pH 7.4, suspended at 2 × 10^8 ^cells/ml in PBS containing 20% glycerol, and stored at -80°C until co-culturing with human immune cells. Quantification of the exponential and stationary phase viable cells before and after freezing showed no significant losses in cell viability (data not shown). Colony forming units (CFUs) were determined by plating serial dilutions of the cultures on MRS agar (data not shown).

### Peripheral blood mononuclear cells assay

This study was approved by Wageningen University Ethical Committee and was performed according to the principles of the Declaration of Helsinki. Peripheral blood of healthy donors was from the Sanquin Blood Bank, Nijmegen, The Netherlands. Before sample collection, a written informed consent was provided. Peripheral blood mononuclear cells (PBMCs) were separated from the blood of healthy donors using Ficoll-Paque Plus gradient centrifugation according to the manufacturer's protocol (Amersham biosciences, Uppsala, Sweden). After centrifugation the mononuclear cells were collected, washed in Iscove's Modified Dulbecco's Medium (IMDM) + glutamax (Invitrogen, Breda, The Netherlands) and adjusted to 1 × 10^6 ^cells/ml in IMDM + glutamax supplemented with penicillin (100 U/ml) (Invitrogen), streptomycin (100 μg/ml) (Invitrogen), and 1% human AB serum (Lonza, Basel, Switzerland). PBMCs (1 × 10^6 ^cells/well) were seeded in 48-well tissue culture plates. After an overnight rest at 37°C in 5% CO_2_, 5 μl aliquots of thawed bacterial suspensions at 2 × 10^8 ^CFU/ml were added to the PBMCs (*L. plantarum: *PBMC ratio of 1:1). PBS (5 μl) and LPS (1 μg) served as negative (PBS) and positive (LPS, TLR4 ligand) controls for the stimulation of PBMCs. IL-10 was produced in sufficient amounts for quantification in response to LPS but not to PBS. Similarly, neither LPS nor the PBS buffer stimulated the production of IL-12. To test the capacity of the 42 *L. plantarum *strains to stimulate PBMC cytokine production, PBMCs from 3 different donors were examined (donors A, B, and C). For donors A and B, separate stationary-phase cultures of each *L. plantarum *strain were used. For donor C, both replicate cultures of each *L. plantarum *strain were examined. In PBMC assays comparing responses to *L. plantarum *WCFS1 wild-type and mutant strains, PBMCs from 3 different donors were examined using 4 independent replicate wild-type and mutant *L. plantarum *cultures harvested during exponential-phase and stationary-phase of growth.

Following 24 hr incubation at 37°C in 5% CO_2_, culture supernatants were collected and stored at -20°C until cytokine analysis. This time point was selected for analysis because previous studies showed that IL-12 levels remain unaltered after 4 days of *L. plantarum *incubation with PBMCs. Although IL-10 was shown to increase 2- fold after 4 days of co-incubation with *L. plantarum*, sufficient cytokine amounts were produced after 24 h to permit flow cytometric measurements [61]. No viable bacteria could be cultured and medium acidification was not observed after incubation of *L. plantarum *strains with the PBMCs for 24 h (data not shown). Cytokines were measured using a FACS CantoII flow cytometer (BD Biosciences, Franklin Lakes, New Jersey) and BD Cytometric Bead Array Flexsets (BD Biosciences) for interleukin (IL)-10 and IL-12p70 (henceforth referred to as IL-12) according to the manufacturer's recommendations. Detection limits were 0.13 and 0.6 pg/ml for IL-10 and IL-12 respectively. Concentrations of analytes were calculated with the use of known standards and plotting the sample values against a standard curve in the BD Biosciences FCAP software. Donor-specific variation in cytokine production capacities was taken into account by dividing the cytokine amounts induced by individual *L. plantarum *strains against average cytokine quantities induced by all *L. plantarum *strains for the same donor. These values were then compared to amounts induced by *L. plantarum *WCFS1 and used for gene-trait matching.

### Identification of candidate genes involved in cytokine secretion by gene-trait matching

*L. plantarum *genes with potential roles in modulating of PBMC cytokine production were identified by *in silico *matching using genotype information referenced from the *L. plantarum *WCFS1 genome (also termed gene-trait matching) [45]. Individual *L. plantarum *WCFS1 gene presence or absence scores for the 42 strains were used as putative predictor variables for PBMC induced IL-10, IL-12 and IL-10/IL-12 amounts by regression using the Random Forest algorithm [38]. The "RandomForest" package for R [62] was used with standard parameter settings. *L. plantarum *WCFS1 genes with the highest variable importance measures by the Random Forest method were selected for deletion analysis.

### Construction of *L. plantarum *WCFS1 gene deletion mutants

A previously described *L. plantarum *Δ*lamA *Δ*lamR *mutant was used in this study [40]. Construction of *L. plantarum *lp_1953, lp_2647-2651, lp_0419-0422 and lp_0423 gene deletion mutants was performed as previously described [63] with several modifications. The mutagenesis vectors were generated by a splicing by overlap extension (SOE) procedure [64]. This procedure was designed to expedite mutagenesis vector construction for *L. plantarum *using a single step, blunt-ended cloning and positive selection for transformants based on chloramphenicol resistance. PCR was used to amplify approximately 1 kb of the 5' and 3' regions flanking the genes targeted for deletion (for primer sequences see Table 4). In addition, the *loxP*-*cat*-*loxP *region of pNZ5319 was amplified using primers Ecl-loxR and Pml-loxF (Table 4). For each mutagenesis vector, the amplicons representing the corresponding 2 flanking regions and the *loxP*-*cat*-*loxP *region were mixed in a 1:1:1 molar ratio and used as template in a second PCR reaction with the 5'forward and 3'reverse flanking primers. These PCR reactions resulted in 3 kb amplicons which were cloned into the integration vector pNZ5319 [63] after prior digestion of the vector with SwaI and Ecl136II. Plasmids were transformed into competent cells of *E. coli *JM109 by electroporation as recommended by the manufacturer (Invitrogen). Plasmid DNA was isolated from *E. coli *using Jetstar columns (Genomed GmbH, Bad Oeynhausen, Germany) using the manufacturer's recommended protocol. DNA sequencing (BaseClear, Leiden, The Netherlands) was performed to confirm the integrity of the cloned genes. The resulting plasmids containing the complete gene replacement cassettes were used for mutagenesis [63].

**Table 4:** Primers used in this study.

Primer	**Sequence**^**a**^
LF1953F	5'- TGCCGCATACCGAGTGAGTAG-3'
LF1953R	5'-**CGAACGGTAGATTTAAATTGTTT**ATCAAAAAACACCGTTAATTTGCATC-3'
RF1953F	5'-**GTACAGCCCGGGCATGAG**CGTGGCCATTAGTTGACGAGAC-3'
RF1953R	5'-AACGCCATCGCACTGATGCATC-3'
Ecl-loxR	5'-AAACAATTTAAATCTACCGTTCG-3'
Pml-loxF	5'-CTCATGCCCGGGCTGTAC-3'
LF1953F2	5'-GCAACGGCTGTCAGTAACCTGCCTTC-3'
RF1953R2	5'-TCAAATCTCGAAGCGGTTCAAAACTG-3'
LF2647F	5'-**GTACAGCCCGGGCATGAG**GGTATTTAGCGAAATATACAGATTG-3'
LF2647R	5'-CTTTAGCCGTCTCATTAGTCG-3'
RF2651F	5'-GGATTACCAAAACGAACATGG-3'
RF2651R	5'-**CGAACGGTAGATTTAAATTGTTT**ACTAGCCATTTTGTTTTTATCTCC-3'
LF2647R2	5'-TGACATGACTATCCTGACTTGC-3'
RF2651F2	5'-AACGTTCAACGGCAGATAAGCC-3'
LF423F	5'-AATTGATACATGTGGTTTCGAAAG-3'
LF423R	5'-**CGAACGGTAGATTTAAATTGTTT**CCAATGCATACTTGTACTCCC-3'
RF423F	5'-**GTACAGCCCGGGCATGAG** CGACTTGATCAATAGCTGAGGG-3'
RF423R	5'-TTGGTTGCCTTGATCGTGTAAG-3'
LF423F2	5'-CTTCAGTTATCGCTACAATCAACG-3'
RF423R2	5'-ACTAACGTACTTTGCACCACGG-3'
LF419F	5'**-GTACAGCCCGGGCATGAG**GACGAGTAATCATCCATTCTGA-3'
LF419R	5'-ATGAGTTTGCAATGGAGCTTAGG-3'
RF422F	5'-CAAAGACGTGCCGAATATAGCC-3'
RF422R	5'-**CGAACGGTAGATTTAAATTGTTT**AAACTGTAGCATAAATAATCCCC-3'
LF419R2	5'-GAGATAATTATTGTAAGACCGTC-3'
RF422F2	5'-CTAACGCATCAATAATCTTACTGG-3'

^**a **^Bold and underlined nucleotides signify overlapping ends with the Ecl-loxR and Pml-loxF primers.

### Statistical analysis

Linear mixed effect models using restricted maximum likelihood (REML) were used to statistically compare the mean cytokine values of IL-10, IL-12, and IL-10/IL-12 produced in response to *L. plantarum *wild-type and mutant cells. The effect of the donor on the response variable was modeled as a random effect. The fixed effects in the model were the strains (WCFS1 [wild type], Δ*pts19ADCBR*, Δlp_1953, Δ*plnG*, Δ*plnEFI*, and Δ*lamA *Δ*lamR*) and the growth phase at the time of harvest (exponential phase and stationary phase). Logarithmic transformations of [IL-10], [IL-12] and [IL-10]/[IL-12] yielded residuals that showed approximately normal distributions (data not shown) and, hence, were used as the response variables in the fitting procedure. Statistical analysis was performed using R http://www.r-project.org, with the package "nlme" [65] for mixed effect modeling. The donor random effect was modeled as a constant offset relative to the average level of the response variable ("model 1"), or alternatively, as a donor-dependent offset plus a donor-dependent variation in the effect of the bacterial growth phase on the response variable ("model 2"). Model 2 yielded better fits for ^2^log([IL-10]) and ^2^log([IL-10]/[IL-12]) response variables whereas, indications of a donor dependent variation in growth phase effects were not found for the ^2^log([IL-12]) response, and hence model 1 was applied for comparison of these cytokine amounts. The resulting relative difference coefficients and t tests were calculated from the fixed effects (mutation, growth phase, and their interaction) using analysis of variance in R. The p-values were adjusted for multiple hypothesis testing using the correction procedures by Hochberg [66].

## Authors' contributions

SvH performed the PBMC assays, constructed the deletion mutants and prepared the manuscript. MM assisted with isolation of PBMCs and flow cytometry for cytokine analysis. DM performed the statistical analysis and gene-trait matching. PB designed the mutagenesis strategy. PdV coordinated the research groups involved in the study and assisted in data interpretation and analysis. MK assisted with the design of the study and help draft the manuscript. JMW helped draft the manuscript, assisted with the design of the study, and supervised a portion of the research. MLM designed the study, supervised a portion of the research, and prepared the manuscript. All authors read and approved the final manuscript.
